# Phase angle, extracellular to intracellular water ratio, and advanced glycation end products according to four sarcopenia categories and in relation to muscle strength/mass in elderly out-patients with diabetes

**DOI:** 10.1007/s13340-026-00894-6

**Published:** 2026-06-05

**Authors:** Toshihiro Amagawa, Hiroki Yokoyama, Yumiko Teramoto, Youko Fujimaki, Atsushi Ohno, Hiroshi Takamura

**Affiliations:** 1 Department of Diabetology and Endocrinology and Metabolism, Tokyo Medical University Hachioji Medical Center, Tatemachi, Hachioji, 1163 Japan; 2 Takamura Clinic, Fussa, 1044 Japan; 3 Internal Medicine, Jiyugaoka Medical Clinic, West 6, South 6-4-3, Obihiro, 080-0016 Japan

**Keywords:** Sarcopenia, Diabetes, Phase angle, Extracellular water/intracellular water ratio, Advanced glycation end products

## Abstract

**Objective:**

Sarcopenia is classified into normal, presarcopenia, dynapenia, and sarcopenia according to muscle strength/mass. As assessment methods for muscle strength and mass, phase angle (PhA), extracellular water/intracellular water ratio (ECW/ICW), and advanced glycation end products (AGEs) have been developed. We investigated the proportions in elderly outpatients with diabetes and elucidate clinical characteristics, including PhA, ECW/ICW, and AGEs among the four groups.

**Methods:**

A cross-sectional study was performed in 695 patients with diabetes aged 65 years or older, who were classified according to the classification proposed by the Asian Working Group for Sarcopenia. Clinical characteristics of the four groups were compared by ANOVA. Associations of grip strength and skeletal muscle index (SMI) with PhA, ECW/ICW, and AGEs and interrelations among grip strength, SMI, PhA, ECW/ICW, and AGEs were analyzed by multiple linear regression analyses.

**Results:**

Proportions of those (male/female, %) classified as normal, presarcopenia, dynapenia, and sarcopenia were 42.3/42.9, 30.3/26.6, 19.9/21.0, and 7.4/9.4, respectively. In order from normal, presarcopenia, dynapenia, and sarcopenia, age, ECW/ICW, and AGEs increased, while grip strength, gait speed, and PhA decreased (*p* < 0.001). Grip strength had significant associations with PhA, ECW/ICW, and AGEs independent of the effect of age, sex, and SMI. SMI had significant associations with PhA and ECW/ICW, but not AGEs. ECW/ICW was independently associated with both grip strength and SMI, together with PhA and AGEs.

**Conclusions:**

PhA, ECW/ICW, and AGEs were significantly different according to the four sarcopenia groups, and ECW/ICW was independently associated with both grip strength and SMI.

## Introduction

Several studies have reported an accelerated loss of skeletal muscle strength and mass in individuals with diabetes compared with their counterparts [1, 2]. The loss of skeletal muscle strength and mass, known as sarcopenia, is strongly associated with an increased risk of adverse outcomes, including frailty, physical disability, and mortality [3–5]. Numbers of patients with diabetes accompanied by sarcopenia are increasing with extended life-expectancy, leading to increasing health care use in real-world diabetes practice [6–9]. Therefore, prevention and early detection of sarcopenia are essential in diabetes outpatient care.

Recently, the Asian working group for sarcopenia (AWGS) proposed a classification of sarcopenia as normal, presarcopenia, dynapenia, and sarcopenia, defined by muscle function/muscle mass as normal/normal, normal/low, low/normal, and low/low, respectively [10]. The classification indicates that muscle function (strength) decline and muscle mass reduction may develop independently [11–13]. However, the proportion and clinical features of elderly outpatients with diabetes in the four groups have yet to be fully elucidated, and a pathophysiological approach to muscle function decline and muscle mass reduction is important for a comprehensive understanding of sarcopenia.

As assessment methods for muscle quality (strength) and quantity (mass), measurements of phase angle (PhA), extracellular water/intracellular water ratio (ECW/ICW), and advanced glycation end products (AGEs) have been developed. PhA is a parameter measured by bioelectrical impedance analysis, which reflects cell membrane structure and integrity [14]. Previous studies showed that PhA was associated with muscle strength and mass, sex, age, nutritional status, and mortality [15, 16]. ECW and ICW in skeletal muscle reflect the extracellular spaces filled with plasma and interstitial fluid, and the muscle cell mass, respectively; thus, an increase in ECW/ICW can indicate an expansion of the non-contractile component relative to muscle cell mass [17]. Previous studies showed an association of ECW/ICW with age, muscle strength, and physical activity [17]. AGEs are sugar-modified adducts which arise during non-enzymatic glycoxidative stress, and can form crosslinks between collagen fibers in intramuscular connective tissues, causing muscle rigidity and loss of elasticity [18, 19]. Using the fluorescent properties of several AGEs, an AGE reader can measure skin autofluorescence and estimate tissue AGSs non-invasively [18]. Such measured AGEs were associated with muscle strength, mass, and physical function [19]. Taken together, the above three tests may help assess the pathophysiology of sarcopenia.

The aim of the study was to investigate the following cross-sectionally in elderly outpatients with diabetes aged 65 years or older: (1) proportions of the four sarcopenia categories in males and females, (2) differences among the four groups in clinical characteristics, including age, grip strength, gait speed, skeletal muscle index (SMI), body mass index (BMI), glycosylated hemoglobin (HbA1c), PhA, ECW/ICW, and AGEs, and (3) how the clinical variables are interrelated, especially how PhA, ECW/ICW, and AGEs are associated with grip strength and SMI.

## Methods

### Patient recruitment

Consecutive patients with diabetes aged 65 years or older who visited Takamura Clinic of Internal Medicine from January 2022 to October 2023 were enrolled in the present study after excluding those meeting the following criteria: severe cognitive impairment, severe psychiatric impairment, and severe cardiac, pulmonary, or musculoskeletal disorders. In addition, those with artificial implants, such as cardiac pacemakers and joints, which did not permit the measurements of bioelectrical impedance, were excluded. The aim of the study, the study protocol, and chances to refuse the participation were informed to all patients, and opt-out system, which was based on the national ethical guidance, was employed in this study. The study was conducted in accordance with the guidelines proposed in the Declaration of Helsinki, and the study protocol was approved by the Ethics Committee of Ichiyoukai on December, 2021 (#211,203).

### Classification of sarcopenia

Patients were divided into four groups: normal, presarcopenia, dynapenia, and sarcopenia, according to the sarcopenia diagnostic criteria from AWGS 2019 based on the results of muscle function decline and SMI measurements [10]. Muscle function decline was defined as grip strength of < 28 kg and/or normal walking speed of < 1.0 m/s for men, and a grip strength of < 18 kg and/or normal walking speed of < 1.0 m/s for women. Muscle mass reduction was defined as SMI < 7.0 kg/m^2^ for men and SMI < 5.7 kg/m^2^ for women. Normal, presarcopenia, dynapenia, and sarcopenia were defined by muscle function/muscle mass as normal/normal, normal/low, low/normal, and low/low, respectively.

### Measurements

Type 2 diabetes was diagnosed according to the Japan Diabetes Society criteria; fasting blood glucose of 7.0 mmol/L (126 mg/dL) or greater, casual blood glucose of 11.1 mmol/L (200 mg/dL) or greater, and HbA1c ≥ 6.5%. HbA1c was measured by high-performance liquid chromatography, which has been certified by the National Glycohemoglobin Standardization Program. Treatment modalities were divided into no drugs, use of non-insulin drugs, and use of insulin with or without use of non-insulin drugs. Serum and urinary concentrations of creatinine (Cr) and urinary albumin were measured by enzymatic methods and turbidimetric immunoassay, respectively. The urinary albumin excretion rate (UAE) was recorded as the albumin-to-creatinine ratio (mg/g Cr). The estimated glomerular filtration rate (eGFR) was calculated using the following equation by the Japanese Society of Nephrology: eGFR (mL/min/1.73 m^2^) = 194 × Scr^−1.094^ × Age^−0.287^ × 0.739 (if female). A previous history of cardiovascular disease (CVD), including coronary artery disease and stroke, was investigated by medical records.

Hand grip strength was measured using Jamar Hand Dynamometer (TKK-5401; Takei Kiki Kougyou Co., Ltd., Niigata, Japan) once for both hands. Patients were encouraged to exert maximal grip strength, and the maximum value was used for analyses. Gait speed was measured by walking a distance of 7m with a 1m acceleration and a 1 1m deceleration after being instructed to walk at a regular speed which was usual for the patient. The length of time to walk 5m (1-6m) was measured.

BMI, ICW, ECW, total body water, lean body mass, body fat percentage, skeletal muscle mass, and PhA were measured by a whole-body bioelectrical impedance data (InBody 770, Tokyo, Japan). This acquisition system consists of the multifrequency and eight-polar tactile-electrode impedance method, and both its accuracy and reproducibility have been well-established elsewhere compared with isotope dilution [20]. PhA reflects cellular membrane integrity and muscle quality, defined by the following equation: PhA (°) = arctangent (reactance/resistance) × (180°/π), where reactance and resistance of 50 kHz were used to calculate PhA (normal range, > 5.04° for male and > 4.20° for female) [14, 21, 22]. The ECW/ICW ratio and SMI (kg/m^2^) were calculated from the obtained data. The normal range (%, 95% intervals) of ECW/ICW ratio in Japanese adults aged 70–79 years was 62.6 (59.7 to 65.4) for male and 63.7 (60.9–66.4) for female, respectively [23].

AGEs were measured by AGE Reader mu (DiagnOptics Technologies BV, Groningen, The Netherlands), which was developed to assess skin autofluorescence (SAF) non-invasively using the fluorescent properties of several AGEs (normal range, 2.5 ± 0.6) [18, 24]. SAF was measured on the volar side of the forearm.

### Statistical analysis

Continuous data are expressed as the mean ± SD if normally distributed variables and the median (interquartile range) if non-normally distributed. Non-normally distributed variables, such as UAE, were first logarithmically transformed before any analysis. Comparisons of continuous variables among the four groups were performed by one-way analysis of variance (ANOVA), and pairwise post-hoc multiple comparisons with a normal group were performed by Tukey’s honestly significant difference test. The significance of differences between groups was assessed by chi-squared tests for categorical variables. Spearman’s rank-sum correlation coefficient was used to examine the simple relationship between two clinical variables. Multiple linear regression analysis was conducted to explore the associations of grip strength and SMI with PhA, ECW/ICW, and AGEs after adjustment for age and sex (Model 1), and for age, sex and SMI (or grip strength) (Model 2). As a final model, the independent determinants of grip strength, SMI, PhA, ECW/ICW, and AGEs were investigated with all covariates including grip strength, SMI, phase angle, ECW/ICW, AGEs, age, and sex being entered in the model. To assess the problems caused by multi-collinearity in the final model, variance inflation factor was investigated as an index. When a variable had collinearity in the model, the variable was excluded from the model and correlation coefficients were recalculated. The partial standardized correlation coefficients (β) were given. A *p-*value less than 5% (two-tailed) was considered significant. All analyses were performed using the SPSS statistical software package (SPSS Japan, Tokyo, Japan).

## Results

There were 695 patients with diabetes (376 males and 319 females; 673/22 with type 2/type 1 diabetes, respectively) who entered the study. Table 1 shows the clinical characteristics of patients according to four sarcopenia categories. Proportions of patients classified to normal, presarcopenia, dynapenia, and sarcopenia were 42.3, 30.3, 19.9, and 7.4% in males, and 42.9, 26.6, 21.0, and 9.4% in females, respectively, where the difference between male and female was not significant. One-way ANOVA indicated that clinical variables including lean body mass, body fat percentage, age, and BMI were significantly different in the four sarcopenia groups, but HbA1c and eGFR values were weakly or not significantly different. Compared with the normal group, PhA was significantly lower in presarcopenia, dynapenia, and sarcopenia groups, and ECW/ICW and AGEs were significantly higher in dynapenia and sarcopenia groups.

**Table 1:** Clinical characteristics of patients with diabetes aged 65 years or older according to the four sarcopenia categories, including PhA, ECW/ICW, and AGEs

	Normal	Presarcopenia	Dynapenia	Sarcopenia	*p*-value
Male, N (%)	159 (42.3%)	114 (30.3%)	75 (19.9%)	28 (7.4%)	
Grip strength (kg)	35.7 ± 4.6	29.7 ± 5.2 ^***^	29.1 ± 6.3 ^***^	22.7 ± 4.1 ^***^	< 0.001
Gait speed (m/sec)	1.27 ± 0.2	1.16 ± 0.2 ^***^	0.93 ± 0.2 ^***^	0.83 ± 0.1 ^***^	< 0.001
SMI (kg/m^2^)	7.6 ± 0.5	6.5 ± 0.3 ^***^	7.5 ± 0.4	6.2 ± 0.5 ^***^	< 0.001
Leam body mass (kg)	51.0 ± 4.3	42.8 ± 2.8^***^	49.1 ± 4.3^**^	39.9 ± 2.6^***^	< 0.001
Body fat percentage (%)	23.6 ± 5.5	23.3 ± 6.2	27.8 ± 6.1^***^	27.6 ± 7.8^**^	< 0.001
Age (years)	72.4 ± 5.2	74.7 ± 5.7 ^**^	76.3 ± 5.5 ^***^	79.1 ± 4.9 ^***^	< 0.001
BMI (kg/m^2^)	23.8 ± 2.4	21.2 ± 2.1 ^***^	25.0 ± 2.4 ^**^	22.0 ± 2.6 ^**^	< 0.001
Duration of diabetes (years)	19.5 ± 9.7	21.7 ± 12.3	20.4 ± 11.3	21.7 ± 12.2	0.495
Diet/non-insulin/insulin (%)	8.8/71.6/17.6	5.3/75.4/19.3	6.7/68.0/25.3	7.1/67.9/25.0	0.741
HbA1c (%)	7.2 ± 0.7	7.2 ± 0.8	7.4 ± 0.9	7.5 ± 1.0	< 0.05
eGFR (ml/min/1.73m^2^)	67.3 ± 17.8	66.7 ± 16.1	61.7 ± 17.3	66.0 ± 18.3	0.135
UAE (mg/g Cr)	11.0(4.2–26.5)	9.6(4.0–19.6)	12.6(6.6–34.1)	14.3(4.4–38.4)	0.075
Past history of CVD (%)	4.4	5.3	10.7	10.7	0.215
PhA (°)	5.1 ± 0.6	4.7 ± 0.6 ^***^	4.6 ± 0.6 ^***^	4.3 ± 0.6 ^***^	< 0.001
ECW/ICW (%)	64.0 ± 2.1	64.2 ± 1.8	65.7 ± 2.3 ^***^	65.7 ± 1.7 ^***^	< 0.001
AGEs (SAF)	2.8 ± 0.5	3.0 ± 0.5	3.1 ± 0.6 ^***^	3.2 ± 0.6 ^**^	< 0.001

Female, N (%)	137 (42.9%)	85 (26.6%)	67 (21.0%)	30 (9.4%)	
Grip strength (kg)	22.4 ± 2.6	18.8 ± 3.5 ^***^	17.9 ± 4.1 ^***^	14.8 ± 2.2 ^***^	< 0.001
Gait speed (m/sec)	1.26 ± 0.2	1.21 ± 0.2	0.94 ± 0.3 ^***^	0.78 ± 0.2 ^***^	< 0.001
SMI (kg/m^2^)	6.3 ± 0.5	5.2 ± 0.3 ^***^	6.2 ± 0.4	5.0 ± 0.5 ^***^	< 0.001
Leam Body mass (kg)	37.9 ± 3.0	32.3 ± 2.5^***^	36.3 ± 3.4^**^	30.7 ± 2.1^***^	< 0.001
Body fat percentage (%)	32.4 ± 7.3	29.8 ± 6.4^**^	35.7 ± 6.9^**^	30.7 ± 8.4	< 0.001
Age (years)	73.2 ± 4.9	75.3 ± 5.6 ^*^	77.6 ± 6.3 ^***^	81.3 ± 6.8 ^***^	< 0.001
BMI (kg/m^2^)	24.1 ± 3.3	20.6 ± 2.3 ^***^	25.1 ± 3.2	21.1 ± 3.2 ^***^	< 0.001
Duration of diabetes (years)	20.1 ± 10.5	21.1 ± 10.0	20.2 ± 10.3	25.3 ± 10.1	0.236
Diet/non-insulin insulin (%)	7.3/70.8/21.9	9.4/69.4/21.2	3.0/65.7/31.3	6.7/73.3/20.0	0.571
HbA1c (%)	7.4 ± 1.2	7.4 ± 1.5	7.5 ± 1.3	7.8 ± 1.4	0.838
eGFR ml/min/1.73m^2^)	66.3 ± 14.9	67.7 ± 15.9	59.5 ± 16.8	67.0 ± 19.7	0.019
UAE (mg/g Cr)	8.7 (4.0–16.6)	8.6 (4.3–16.8)	12.2 (5.8–31.5)	9.2 (6.5–15.9)	0.263
Past history of CVD (%)	2.2	3.5	4.5	3.0	0.838
PhA (°)	4.5 ± 0.5	4.1 ± 0.4 ^***^	4.2 ± 0.5 ^***^	3.6 ± 0.5 ^***^	< 0.001
ECW/ICW (%)	65.0 ± 1.7	65.6 ± 1.7	66.6 ± 2.4 ^***^	68.0 ± 2.2 ^***^	< 0.001
AGEs (SAF)	2.6 ± 0.4	2.7 ± 0.5	2.7 ± 0.5	3.0 ± 0.5 ^***^	< 0.001

*p*-values by one-way ANOVA are given. ^*^p < 0.05, ^**^p < 0.01, and ^***^p < 0.001 versus normal obtained from pairwise post-hoc multiple comparisons by Tukey’s honestly significant difference test

SMI, Skeletal muscle index; BMI, Body mass index, HbA1c, Glycosylated hemoglobin; eGFR, Estimated glomerular filtration rate, UAE: urinary albumin excretion rate, CVD: cardiovascular disease, PhA: phase angle, ECW/ICW: extracellular water/intracellular water ratio, AGEs: advanced glycation end products, SAF: skin autofluorescence

A simple correlation between two clinical variables is shown by Spearman’s correlation coefficients in Table 2. An older age was significantly correlated with female predominance, lower levels of grip strength, gait speed, SMI, and PhA, and higher levels of ECW/ICW and AGEs. Female (vs. male) was associated with lower levels of grip strength, SMI, PhA, and AGEs, and higher levels of ECW/ICW. Higher BMI was correlated with higher levels of grip strength, SMI, and PhA, and lower levels of gait speed. Higher grip strength was correlated with higher levels of gait speed, SMI, and PhA, and lower levels of ECW/ICW. Higher gait speed was correlated with higher levels of SMI and PhA, and lower levels of ECW/ICW and AGEs. Higher SMI was correlated with higher levels of PhA and AGEs, and lower levels of ECW/ICE. Higher PhA was correlated with lower levels of ECW/ICW and AGEs. Higher ECW/ICW was correlated with higher levels of AGEs.

**Table 2:** Spearman’s correlation coefficients between two variables to indicate a simple correlation

	Age	F vs. M	BMI	Grip strength	Gait speed	SMI	PhA	ECW/ICW	AGEs
Age	–	0.084^*^	0.011	− 0.289 ^***^	− 0.389 ^***^	− 0.201 ^***^	-0.413 ^***^	0.470 ^***^	0.218 ^***^
F versus M	–	–	0.031	− 0.762 ^***^	− 0.004	− 0.715 ^***^	-0.427 ^***^	0.279 ^***^	− 0.239 ^***^
BMI	–	–	–	0.112 ^***^	− 0.153 ^***^	0.463 ^***^	0.157 ^***^	0.039	− 0.035
Grip strength	–	–	–	–	0.247 ^***^	0.767 ^***^	0.596 ^***^	− 0.428 ^***^	0.043
Gait speed	–	–	–	–	–	0.100 ^**^	0.338 ^***^	− 0.391 ^***^	− 0.185 ^***^
SMI	–	–	–	–	–	–	0.525 ^***^	− 0.243^***^	0.102 ^**^
PhA	–	–	–	–	–	–	–	− 0.862 ^***^	− 0.129 ^***^
ECW/ICW	–	–	–	–	–	–	–	–	0.202 ^***^

^*^p < 0.05, ^**^p < 0.01, and ^***^p < 0.001

F, Female; M, Male, BMI, Body mass index; SMI, Skeletal muscle index; PhA, Phase angle; ECW/ICW, Extracellular water/intracellular water ratio; AGEs Advanced glycation end products

Table 3 shows associations of grip strength, gait speed, and SMI with PhA, ECW/ICW, and AGEs adjusted for a) age and sex, and b) age, sex, and SMI (or grip strength) in multiple linear regression analyses. Both grip strength and gait speed had significant associations with PhA, ECW/ICW, and AGEs independent of the effect of age, sex, and SMI. SMI showed significant associations with PhA in both models, in which the significance with ECW/ICW and AGEs depended on the models.

**Table 3:** Associations of grip strength, gait speed, and SMI with PhA, ECW/ICW, and AGEs adjusted for a) age and sex (Model 1), and b) age, sex, and SMI (or grip strength) (Model 2) in multiple linear regression analyses

		a) adjusted for age and sex (Model 1)			b) adjusted for age, sex, and SMI (Model 2)		
		β	*p*-value	R^2^	β	*p*-value	R^2^
Grip strength	PhA	0.282	< 0.001	0.643	0.192	< 0.001	0.696
	ECW/ICW	− 0.152	< 0.001	0.606	− 0.177	< 0.001	0.696
	AGEs	− 0.112	< 0.001	0.601	− 0.087	< 0.001	0.680
Gait speed	PhA	0.282	< 0.001	0.204	0.288	< 0.001	0.203
	ECW/ICW	− 0.319	< 0.001	0.222	− 0.236	< 0.001	0.226
	AGEs	− 0.115	< 0.01	0.162	− 0.111	< 0.01	0.163

		a) adjusted for age and sex (Model 1)			b) adjusted for age, sex, and grip strength (Model 2)		
		β	*p*-value	R^2^	β	*p*-value	R^2^
SMI	PhA	0.263	< 0.001	0.545	0.140	< 0.001	0.613
	ECW/ICW	0.060	0.063	0.501	0.141	< 0.001	0.615
	AGEs	− 0.062	0.031	0.502	-0.006	0.820	0.601

β indicates partial standardized correlation coefficient

SMI, Skeletal muscle index, PhA, Phase angle; ECW/ICW, Extracellular water/intracellular water ratio; AGEs, Advanced glycation end products

Table 4 shows multiple linear regression analyses to explore the independent variables which determine grip strength, SMI, PhA, ECW/ICW, and AGEs when all covariates including grip strength, SMI, PhA, ECW/ICW, AGEs, age, and sex were entered in the model. Significant positive correlations were found between grip strength and SMI, SMI and PhA, SMI and ECW/ICW, and ECW/ICW and AGEs. Significant negative correlations were found between grip strength and ECW/ICW, and PhA and ECW/ICW. Correlation coefficients, indicated as a) to e), were recalculated to minimize collinearity problem caused by the close correlations between grip strength and SMI, and between PhA and ECW/ICW.

**Table 4:** Partial standardized correlation coefficients in multiple linear regression analyses to determine grip strength, SMI, phase angle, ECW/ICW, and AGEs when all covariates including grip strength, SMI, phase angle, ECW/ICW, AGEs, age, and sex were entered in the model

Objective variable	Selected variable					R^2^
	Grip strength	SMI	PhA	ECW/ICW	AGEs	
Grip strength	–	0.691^***^	− 0.026^a)^	− 0.299^***^	− 0.032	0.626
SMI	0.576^***^	–	0.784 ^***^	0.686^***^	− 0.035	0.688
PhA	− 0.010^b)^	0.357 ^***^	–	− 0.783^***^	0.011	0.858
ECW/ICW	− 0.142^***^	0.390 ^***^	− 0.977^***^	–	0.043^*^	0.823
AGEs	0.081^c)^	0.103^d)^	− 0.074^e)^	0.224^*^	–	0.068

^*^p < 0.05, ^**^p < 0.01, and ^***^p < 0.001

Correlation coefficients, indicated as a) to e), were recalculated to minimize collinearity problem caused by the close correlations between grip strength and SMI, and between PhA and ECW/ICW

^a)^became 0.277 (*p* < 0.001) when ECW/ICW was excluded from the model because of the collinearity between PhA and ECW/ICW

^b)^became 0.272 (*p* < 0.001) when SMI was excluded from the model because of the collinearity between grip strength and SMI

^c)^became 0.141 (*p* < 0.001) when SMI was excluded from the model because of the collinearity between grip strength and SMI

^d)^became 0.159 (*p* < 0.001) when grip strength was excluded from the model because of the collinearity between grip strength and SMI

^e)^became − 0.296 (*p* < 0.001) when ECW/ICW was excluded from the model because of the collinearity between PhA and ECW/ICW

SMI, Skeletal muscle index, PhA, Phase angle; ECW/ICW, Extracellular water/intracellular water ratio; AGEs Advanced glycation end products

**Fig. 1:**
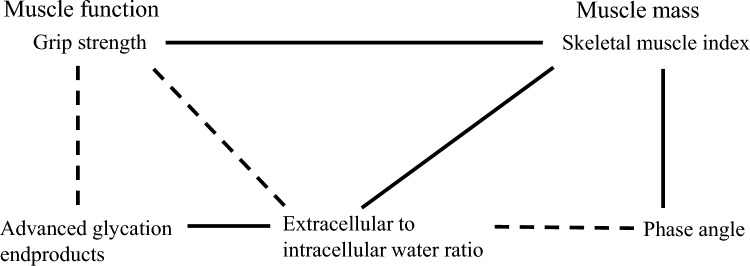
Associations of phase angle, extracellular to intracellular water ratio, and advanced glycation end products with grip strength and SMI, indicated as the independent determinants in multiple linear regression analyses. Solid line indicates positive correlations and dotted line indicates negative correlations

Most of the correlations did not change if grip strength was replaced by gait speed (i.e., another muscle function parameter), while the significance became weaker. Figure 1 shows the positive and negative associations among these five variables.

## Discussion

This study examined elderly outpatients with diabetes in a primary care setting, and found that proportions (male/female, %) of normal, presarcopenia, dynapenia, and sarcopenia were 42.3/42.9, 30.3/26.6, 19.9/21.0, and 7.4/9.4, respectively. PhA, ECW/ICW, and AGEs were significantly different according to the four groups, and significantly correlated with muscle strength independent of the effect of age, sex, and SMI.

To date, only a few studies have examined the proportions according to the four groups. Mori H et al. reported dynapenia at 20.9% and sarcopenia at 11.4% in patients with type 2 diabetes aged 65 years or older visiting large hospitals (with a secondary care setting) [6]. The remainder of 67.7% belonged to those classified as normal or presarcopenia which was similar to our results, while the study did not define presarcopenia. Mori K et al. reported normal at 60.9%, presarcopenia at 25.7%, dynapenia at 5.2% and sarcopenia at 8.1% in community-dwelling older women, i.e., a general population [11]. One follow-up study reported a significantly higher risk of incident sarcopenia in subjects with diabetes than in those without diabetes [25]. These findings suggest that in patients with diabetes, normal is low, and non-normal (sum of presarcopenia, dynapenia, and sarcopenia) is high.

Clinical characteristics by the four sarcopenia groups revealed that, in order from normal, presarcopenia, dynapenia, and sarcopenia, age, ECW/ICW, and AGEs increased while grip strength, gait speed, and PhA decreased. PhA was significantly lower in presarcopenia, dynapenia, and sarcopenia compared with normal. PhA was significantly associated with age, sex, grip strength, gait speed, and SMI. The finding was consistent with a previous report which showed a significant decrease of PhA in dynapenia and sarcopenia and significant associations of PhA with grip strength and gait speed in community-dwelling older adults [14]. We further found that the effect of PhA on grip strength was independent of age, sex, and SMI, and PhA was independently associated with SMI and ECW/ICW. A previous follow-up study indicated an increase of PhA after a resistance training program [26]. A review paper reported that lower PhA values were correlated with higher C reactive protein, tumor necrosis factor-α, and interleukin-6, indicating that PhA has potential utility in early detection of the inflammatory and oxidative status [27]. The above findings suggest that PhA is likely a sensitive marker of muscle strength and mass.

ECW/ICW values were significantly elevated in dynapenia and sarcopenia, but not in presarcopenia compared with normal in both sexes. To our knowledge, this is the first report of ECW/ICW by the four groups of sarcopenia in patients with diabetes. The finding was in line with Iwasaka et al. [17], who showed that ECW/ICW reflected muscle strength more than muscle mass. The association of ECW/ICW with muscle strength was consistent with others observed in community-dwelling older people [17, 28, 29]. Of interest, gait speed was rather lower in dynapenia than in presarcopenia despite the similar grip strength as shown in Table 1. This was concordant with a finding that β values of ECW/ICW with gait speed was two times negatively greater than those with grip strength as shown in Table 3. While dynapenia is characterized by low muscle function and normal muscle mass, we presume that the preserved muscle mass (SMI) in dynapenia may be derived from preserved (or increased) muscle extracellular fluid (non-contractile) and decreased muscle cell mass (contractile). Relative expansion of ECW against ICW in muscle may decrease the quality of muscle (21), leading to lowering gait speed which needs more continuous and overall muscle power as compared with grip strength as observed in Table 1. The contradictory finding that ECW/ICW was negatively associated with grip strength but positively with SMI might be explained by the seemingly preserved SMI. Increase in ECW may inflate apparent SMI, and therefore, skeletal muscle mass, if masked by preserved or increased ECW, may not reflect actual muscle atrophy [30]. This may explain the inconsistency why the relationship between SMI and ECW/ICW was negative in Table 2 affected by aging and sex, but became positive in Table 3 after adjustment for the effect of age, sex, and grip strength. Taken together, we consider that patients with dynapenia had reduced muscle strength and seemingly preserved muscle mass, in which elevated ECW/ICW found in dynapenia may be closely related to the reduction of muscle strength as an important determinant of muscle strength. Alternatively, the reason why ECW/ICW was not decreased in the presarcopenia group may be that ECW and ICW were proportionally decreased in presarcopenia.

AGEs, measured by an AGE reader, showed a significant negative association with grip strength and positive association with ECW/ICW. Our findings are supported by previous studies using the AGE reader, which indicated a significant negative association of skin AGEs with muscle function and muscle mass [31–33]. AGEs accumulation between collagen fibers in intramuscular connective tissues may be associated with more ECW expansion rather than ICW, leading to a decrease in muscle strength. However, because the associations of skin AGEs with muscle AGEs remain within assumption, and correlations with AGEs in the present study were not strong, we should acknowledge that future investigations are needed.

Because age and sex have strong associations with muscle function and muscle mass, we adjusted for the effects of age and sex in multiple linear regression analyses and explored which variables including PhA, ECW/ICW, and AGEs were associated with grip strength and SMI as shown in Table 3. The model indicated independent determinants of grip strength to be PhA, ECW/ICW, and AGEs, and those of SMI to be PhA in both Model 1 and 2 (ECW/ICW and AGEs depended on the model). In the final model when all variables entered, ECW/ICW were independently associated with all variables of grip strength, SMI, PhA, and AGEs, which may reinforce the importance of ECW/ICW in sarcopenia. The association of ECW/ICW with PhA may reflect cell membrane integrity (reactance) and body fluid (resistance) [34], and combined effect of PhA with ECW/ICW increased the odds ratio to predict poor muscle function [35]. Interestingly, a randomized controlled clinical trial demonstrated that intervention by whey protein supplementation with resistance training promoted a reduction in ECW/ICW and resistance training improved PhA in older women [36]. ECW/ICW, together with PhA, may be an intervention marker for sarcopenia.

There are several strengths and limitations that must be addressed in this study. This study included relatively a large number of elderly outpatients with diabetes. For the first time, we reported the proportions by the four sarcopenia groups, and examined which sarcopenia-related tests of PhA, ECW/ICW, and AGEs were dominantly associated with muscle function and muscle mass. These tests would be important for understanding the pathophysiology of sarcopenia. However, the study was only performed in a single institution with Japanese patients. Future studies including other diabetes clinics and non-Japanese populations are necessary. Secondly, the design of the present study was cross-sectional. PhA and ECW/ICW may indicate muscle quality and AGEs may reflect an alteration in muscle health outcomes (strength, mass, and function) (19). It is necessary to investigate changes of these parameters in relation to grip strength and SMI in longitudinal studies. Ideally, we wish if these parameters became useful to prevent progression of sarcopenia after initiating weight-lowering anti-diabetic drugs such as sodium–glucose cotransporter 2 inhibitors and glucagon-like peptide 1 receptor agonists. Thirdly, we should acknowledge the merits and demerits of the three sarcopenia-related tests of PhA, ECW/ICW, and AGEs. All these tests are convenient, non-invasive, easy to perform, and speedy, and so are capable of measuring many subjects in busy outpatient clinics. However, in AGE reader, other non-AGEs fluorescent substances, presence of blood vessels, and skin pigments can interfere with measurements. Bioelectrical impedance analysis is influenced by the hydration status and body composition. Thus, we have to consider these effects when interpreting the results. Finally, regarding PhA and ECW/ICW, we employed whole-body measures as well as other studies [14–16, 26, 28], while some studies showed the usefulness of segmental ones [17, 29, 37]. It remains controversial which one is superior [27, 37], and so further studies are needed.

In conclusion, proportions of AWGS-proposed sarcopenia classifications and clinical features including PhA, ECW/ICW, and AGEs according to the groups were clarified in elderly outpatients with diabetes. PhA, ECW/ICW, and AGEs were closely associated with muscle function and muscle mass, in which ECW/ICW had close associations with considerable variables. Future sarcopenia studies including these tests are warranted.

## Data Availability

The data sets for the current study are available from the corresponding author upon reasonable request.

## Abbreviations

AWGS: Asian Working Group for Sarcopenia
PhA: Phase angle
ECW/ICW: Extracellular water/intracellular water ratio
AGEs: Advanced glycation end products
SMI: Skeletal muscle index
BMI: Body mass index
HbA1c: Glycosylated hemoglobin
Cr: Creatinine
UAE: Urinary albumin excretion rate
eGFR: Estimated glomerular filtration rate
CVD: Cardiovascular disease
ANOVA: Analysis of variance
SAF: Skin autofluorescence
β: Standardized correlation coefficients
